# Incorporating hybrid models into lysine malonylation sites prediction on mammalian and plant proteins

**DOI:** 10.1038/s41598-020-67384-w

**Published:** 2020-06-29

**Authors:** Chia-Ru Chung, Ya-Ping Chang, Yu-Lin Hsu, Siyu Chen, Li-Ching Wu, Jorng-Tzong Horng, Tzong-Yi Lee

**Affiliations:** 1 Department of Computer Science and Information Engineering, National Central University, Taoyuan, 32001 Taiwan; 2 School of Life and Health Sciences, The Chinese University of Hong Kong, Shenzhen, 518172 China; 3 Department of Biomedical Sciences and Engineering, National Central University, Taoyuan, 32001 Taiwan; 4 Department of Bioinformatics and Medical Engineering, Asia University, Taichung, 41359 Taiwan; 5 Warshel Institute for Computational Biology, The Chinese University of Hong Kong, Shenzhen, 518172 China

**Keywords:** Computational models, Machine learning, Protein function predictions

## Abstract

Protein malonylation, a reversible post-translational modification of lysine residues, is associated with various biological functions, such as cellular regulation and pathogenesis. In proteomics, to improve our understanding of the mechanisms of malonylation at the molecular level, the identification of malonylation sites via an efficient methodology is essential. However, experimental identification of malonylated substrates via mass spectrometry is time-consuming, labor-intensive, and expensive. Although numerous methods have been developed to predict malonylation sites in mammalian proteins, the computational resource for identifying plant malonylation sites is very limited. In this study, a hybrid model incorporating multiple convolutional neural networks (CNNs) with physicochemical properties, evolutionary information, and sequenced-based features was developed for identifying protein malonylation sites in mammals. For plant malonylation, multiple CNNs and random forests were integrated into a secondary modeling phase using a support vector machine. The independent testing has demonstrated that the mammalian and plant malonylation models can yield the area under the receiver operating characteristic curves (AUC) at 0.943 and 0.772, respectively. The proposed scheme has been implemented as a web-based tool, Kmalo (https://fdblab.csie.ncu.edu.tw/kmalo/home.html), which can help facilitate the functional investigation of protein malonylation on mammals and plants.

## Introduction

Lysine malonylation (Kmal), a reversible post-translational modifications (PTMs), can be identified by mass spectrometry and database searching^1^. Several studies have indicated that PTMs play critical roles in regulating cellular functions and are related to a lot of disease progression^2–5^. For instance, type 2 diabetes has been reported to be regulated by malonylation, particularly the pathways associated with fatty acid metabolism and the glucose^6^. Both mitochondrial and enzymes urea cycle have been shown to be regulated by protein malonylation^7^. Additionally, it is confirmed that lysine malonylation is a new type of histone PTM and the unusual modification of histones induces diseases, such as cancer^8^. In plants, malonylation has been shown to be a critical reaction in the metabolisms of xenobiotic phenolic glucosides in tobacco and Arabidopsis^9^. Meanwhile, previous experiments targeting lysine malonylaome in *Oryza sativa* and *Triticum aestivum L*. have demonstrated a dominate presence of malonylated proteins in the metabolic processes which include the tricarboxylic acid (TCA) cycle, carbon metabolisms, photosynthesis, and glycolysis/gluconeogenesis^10,11^.

A number of computational methods have been introduced to predict malonylation sites based on machine learning approaches. Xu et al. developed the first web-server, Mal-Lys, to predict Kmal sites for *M. musculus* proteins^12^. Specifically, the support vector machine (SVM) and minimum redundancy maximum relevance (mRMR) technique were adopted to develop the prediction model by considering features from the peptide fragments, including the position specific amino acid propensity, sequence order information, and physicochemical properties. Xiang et al. used pseudo amino acids as features to construct an SVM-based classifier^13^. Wang et al. took multiple organisms into consideration to build a novel online prediction tool, MaloPred, for the identification of malonylation sites in *E.coli*, *M. musculus*, and *H. sapiens*, separately, by integrating not only protein sequence information and physicochemical properties, but also evolutionarily similar features^14^. Taherzadeh et al. further investigated whether different species require different prediction models to maximize the accuracy^15^. By training the models using data from mice and testing them on other species, they found similar underlying physicochemical mechanisms between mice and humans, but not between mice and bacteria. It should be noted that their SVM-based web server, SPRINT-Mal, was the first online malonylation sites prediction tool to take into account the predicted structural properties of the proteins. Zhang et al. provided systematic comparisons of sequence-based features, physicochemical-property-based features, and evolutionary-derived features in the identification of Kmal sites for *E. coli*, *M. musculus*, and *H. sapiens*, respectively^16^. Random forest (RF), SVM, LightGBM, K-nearest neighbor (KNN), and logistic regression (LR) were adopted to generate optimal feature sets. The integration of the single-method-based models through ensemble learning was found to improve the prediction performance in independent tests. Ahmed et al. proposed a new hybrid resampling method for highly imbalanced data^17^. Furthermore, deep learning approach has recently been widely applied to biological sequence analysis^18–21^. Chen et al. first constructed an integration of the deep learning model based on long short-term memory (LSTM) with an RF classifier for the prediction of mammalian malonylation sites^22^. Due to the strong capability of the deep learning methodology to learn sparse representation, this methodology showed a superior performance compared to traditional machine learning model. In addition to the malonylation site, many computational methods have been developed for the prediction of various PTM sites based on protein sequences^23–28^.

To improve our understanding of the mechanism of malonylation, it is necessary to identify the malonylation sites accurately in advance. However, the experimental identification was mainly performed using mass spectrometry, which is time-consuming, labor-intensive, and expensive. Computational approaches could be used to effectively and accurately identify malonylation sites. Currently, existing computational approaches mostly rely on feature engineering, while deep learning is capable of excavating the underlying characteristics from a large-scale training dataset. Additionally, although the biological functions of malonylation in plants require attention, the currently existing tools have only taken into account malonylation sites in humans, mice, and bacteria. An efficient methodology for the identification of malonylation sites in more organisms would greatly improve the understanding of the mechanisms of malonylation. Therefore, the primary purpose of this study was to develop hybrid models combining CNN and machine learning algorithms for the prediction of malonylation sites in mammals and plants, respectively. Meanwhile, a user-friendly web tool, which includes an optimal classifier, was established for individual use in the identification of malonylation sites.

## Results

### Sequence analysis

**Figure 1:**
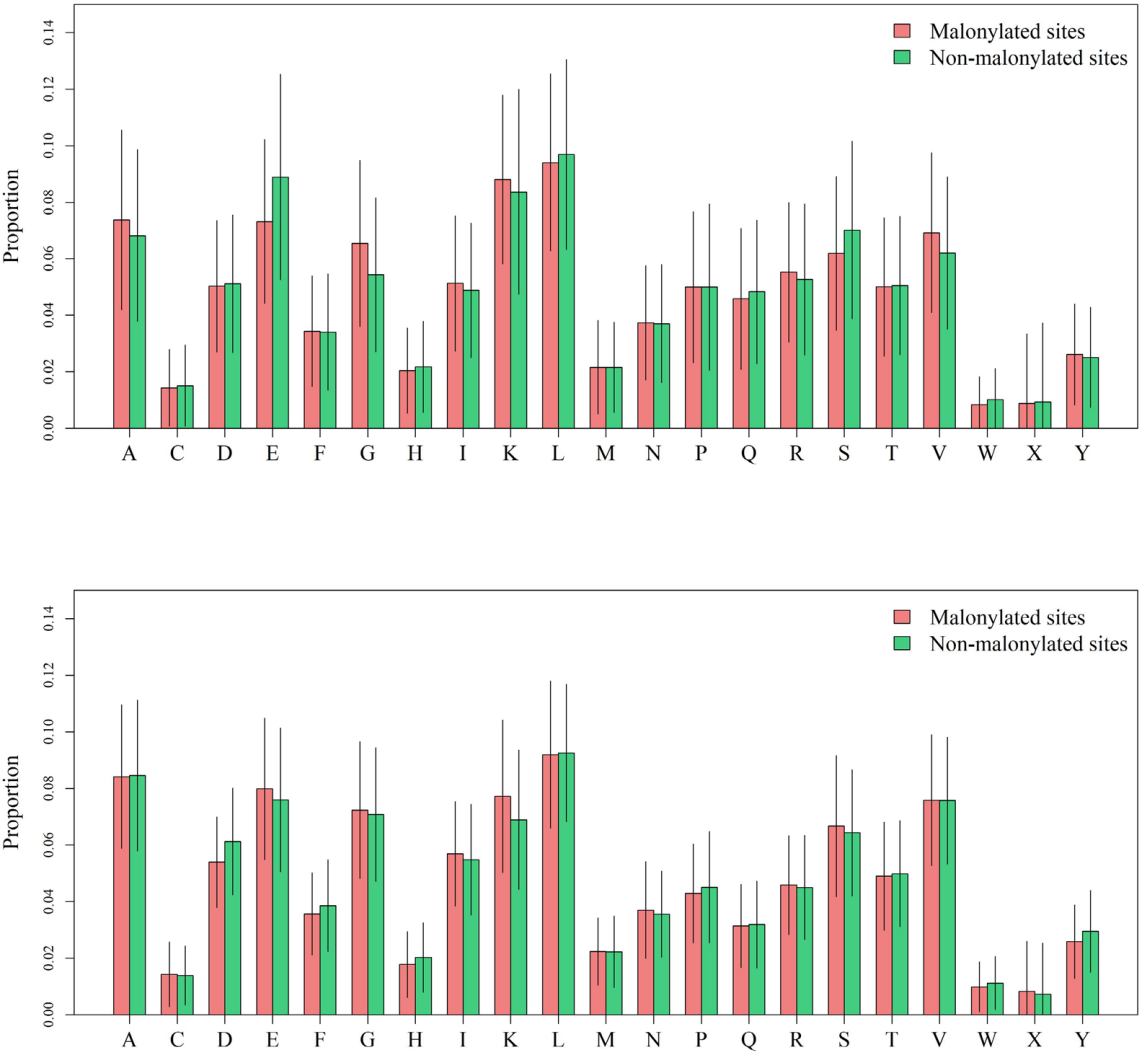
The AACs of malonylation and non-malonylation sites in mammalians (upper) and plants (bottom).

As shown in Fig. 1, the amino acid compositions (AACs) of the malonylation and non-malonylation peptides varied between Glutamic acid (E), Glycine (G), Serine (S), and Valine (V) in mammalian proteins. On the other hand, the AACs in the plant proteins varied between Glutamic acid (E), Aspartic acid (D), Arginine (R), and Tryptophan (W). Only the composition of Glutamic acid (E) varied in both mammalian and plant proteins.

**Figure 2:**
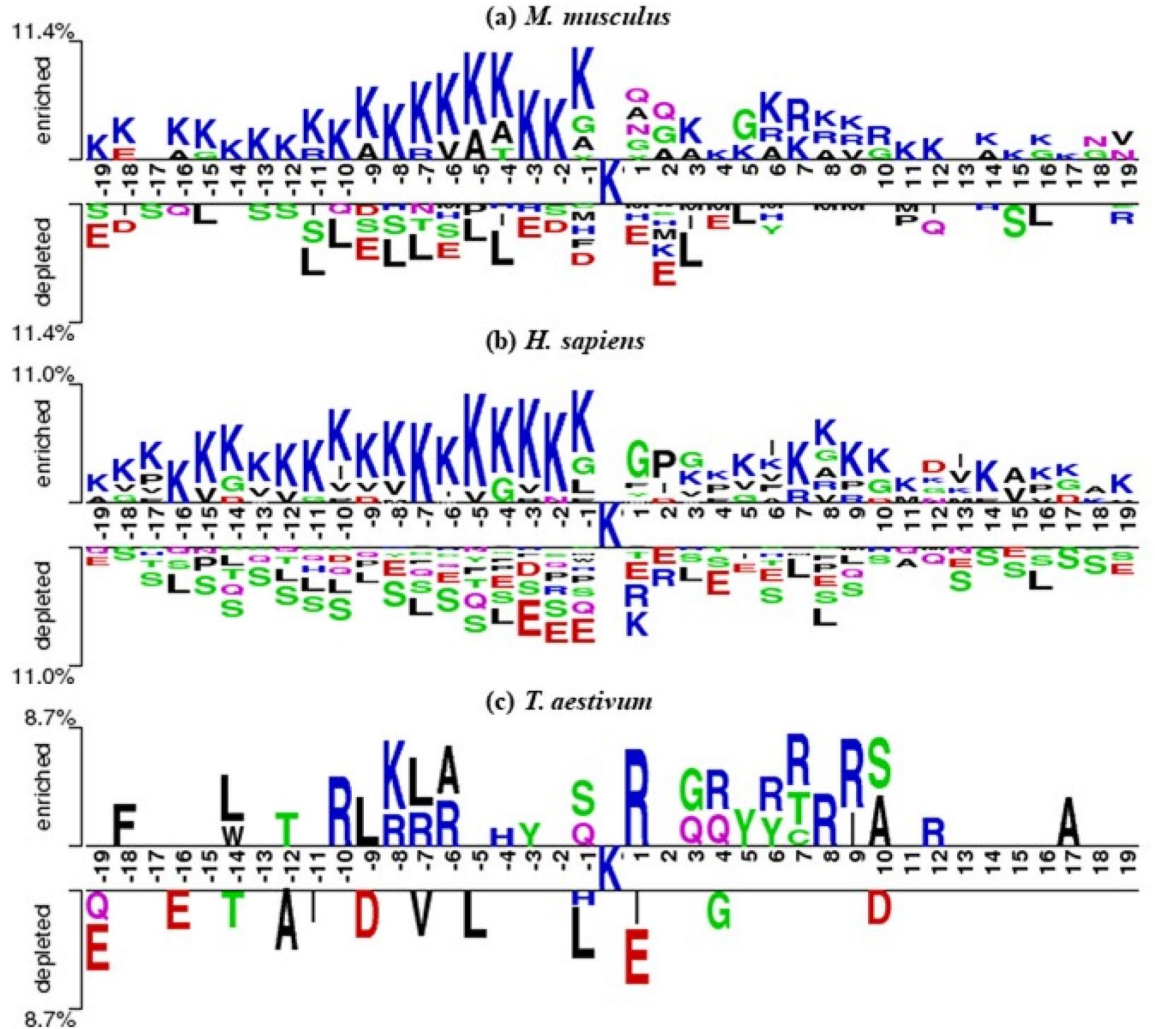
The two sample logo of malonylation sites in (**a**) *H. sapiens*, (**b**) *M. musculus*, and (**c**) *T. aestivum*.

**Figure 3:**
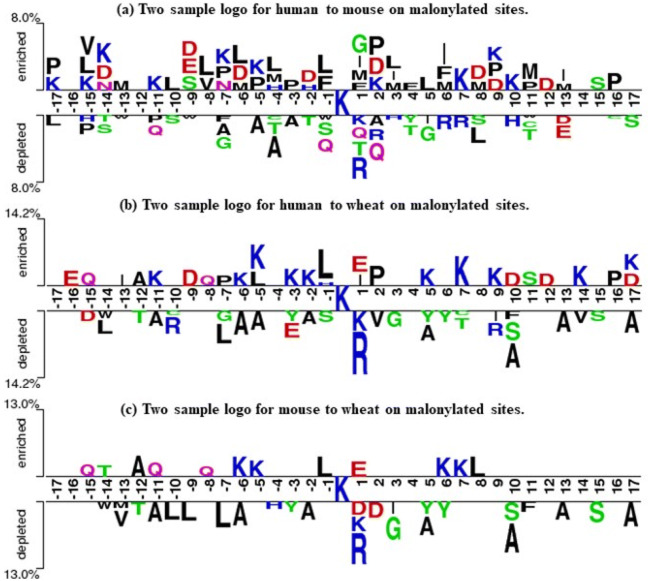
The two sample logo of malonylation sites in (**a**) human to mouse, (**b**) human to wheat, and (**c**) mouse to wheat.

WebLogo was mainly used to analyze the frequencies of occurrence of every position around the malonylation sites^29^. In Supplementary Fig. S1, mouse and human, both mammals, tended to have similar patterns. More specifically, the malonylation peptides had higher frequencies in the cases of Lysine (K), Leucine (L), and Glutamic acid (E) in mammalian proteins. As shown in Fig. 2, using the TwoSampleLogo^30^ of malonylation and non-malonylation peptides, the enrichment of amino acids neighboring the malonylation sites across species was observed. Lysine (K) was found to be significantly enriched at multiple positions in both *H. sapiens* and *M. musculus* proteins, particularly at positions from 1 to 19 and positions 27, 28, and 29. Meanwhile, leucine (L) was found to be depleted at positions 9, 10, and 13. On the other hand, *T. aestivum* proteins showed a very different pattern when compared to *H. sapiens* and *M. musculus* proteins for arginine (R) enrichment at positions 10, 12, 13, 14, 21, 28, and 29 and no evidence of serine (S) depletion. The two sample logo for *H. sapiens* to *M. musculus*, *H. sapiens* to *T. aestivum*, and *M. musculus* to *T. aestivum* is shown in Fig. 3, which indicates that *T. aestivum* is different from the mammals. Subsequently, the data of the *H. sapiens* and *M. musculus* proteins could be combined to build a prediction model for mammals, thereby building another prediction model for *T. aestivum*.

### Feature analysis

Pearson’s correlation coefficient (PCC) was employed to evaluate the dependency between the label and the feature in the training dataset. It should be noted that the PCC values always range from + 1 to –1. A value greater than zero denotes a positive association, whereas a value less than zero denotes a negative association. A value equal to zero indicates that there is no correlation. Therefore, the larger the absolute value of the PCC value, the stronger the correlation.

**Figure 4:**
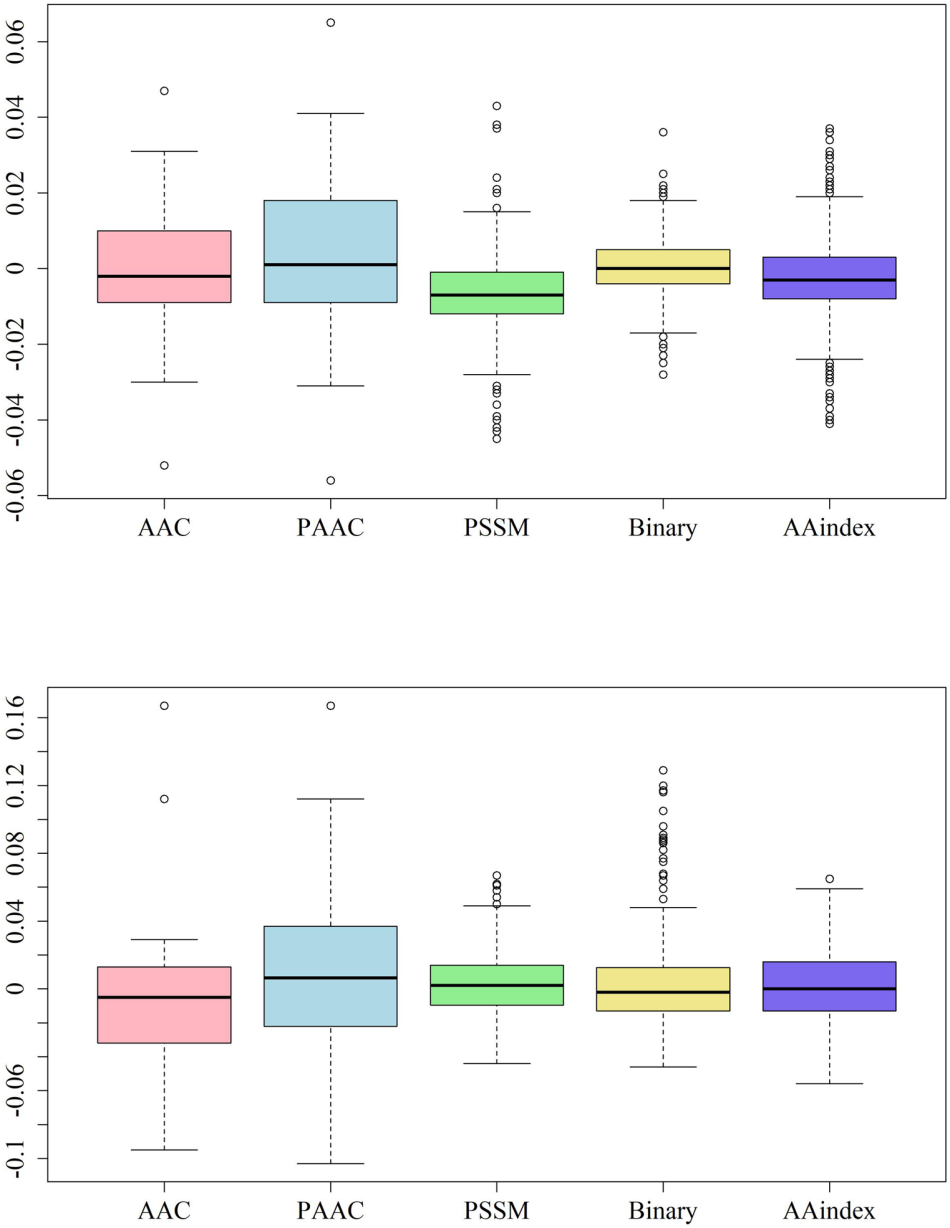
The PCC values of the features in mammalian (upper) and plant (bottom) proteins.

The top ten PCC values of each feature in the mammalian dataset are shown in Supplementary Table S1 and Fig. 4, respectively. The AACs of “E”, “G”, “V”, and “S” are relatively correlated to malonylation, as shown in Fig. 4. Furthermore, the attributes related to amino acids “E”, “G”, and “S” are also found in the top 5 PCC values of the pseudo-amino acid composition (PAAC) group, indicating that these amino acid may be highly correlated with malonylation in mammalian proteins. As for the position-specific features, the top 3 PCC values in the one hot encoding group were as follows: (i) whether the residue next to the central “K” from the downstream was “G”; (ii) whether the residue next to the central “K” from the downstream was also “K”; (3) whether the residue next to the central “K” from the upstream was “G”. All three attributes focus on the position next to the central “K”. The same position tendency can be observed in the AAindex group. All of the highest PCC values focused on position 18 (the residue adjacent to central “K” in the downstream position). In the position-specific scoring matrix (PSSM) group, position 13 (the 4th residue adjacent to the central “K” in the upstream position) had higher PCC values.

In terms of plant proteins, the top ten PCC values of each feature are shown in Supplementary Table S2, and Fig. 4. The listed features are very different from those of the mammalian proteins. The amino acids “R”, “D”, and “W” are only relatively correlated to the plant protein labels. The amino acid “E” is the only one that appears in both organism groups. The positional importance in the plant group is not significant compared to the mammalian group. However, the top PCC values in the AAindex group indicate that malonylation may be positively correlated with side chain hydropathy (ROSM880102) and the transfer of free energy from oct to wat (RADA880102).

### Functional analysis

**Figure 5:**
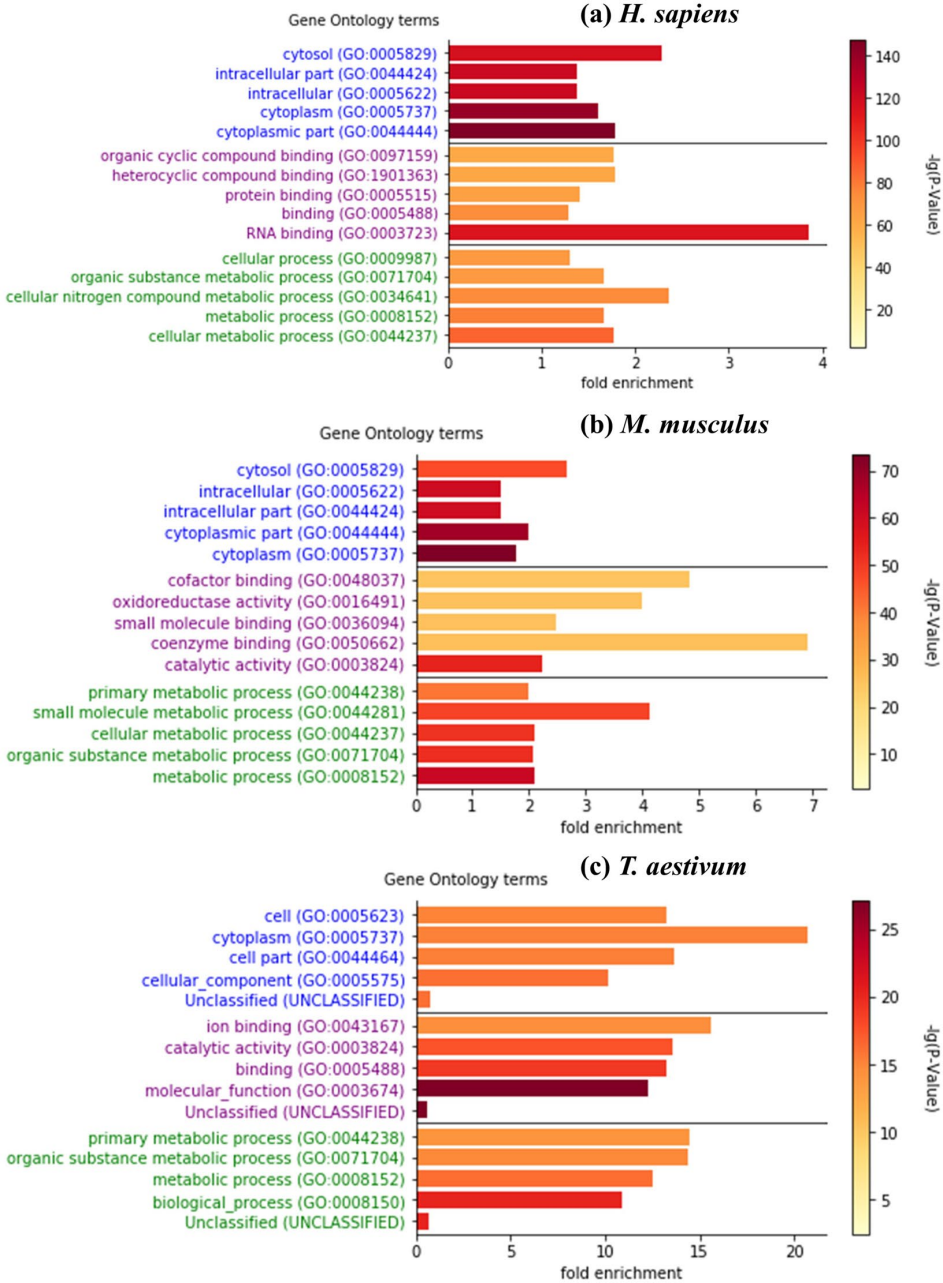
The functional distributions of malonylated proteins for (**a**) *H. sapiens*, (**b**) *M. musculus*, and (**c**) *T. aestivum*, including GO terms in biological process, molecular functions and cellular components.

We used the classification system PANTHER to analyze the functional distribution of malonylated proteins^31^. Figure 5 shows that *H. sapiens* and *M. musculus* shared a highly common functional distribution in terms of biological processes. Both were statistically enriched in the cytosol (GO:005829), intracellular (GO:0005622), intracellular part (GO:00044424), cytoplasm (GO:0005737), and cytoplasmic part (GO:0044444). In terms of the cellular components, both *H. sapiens* and *M. musculus* were statistically enriched in the cellular metabolic process (GO:0044237). The common processes in which all three species were statistically enriched were the cytoplasm (GO:005737) and the organic substance metabolic process (GO:0071704).

### Determination of the window size and the model of each feature

Supplementary Fig. S2, shows the AUC performance of each feature with a different window size of peptides. For the mammalian model, the feature AAINDEX, one hot vector, and PSSM resulted in the best AUC using CNN models with a window size of 37, 33, and 35, respectively. Feature AAC and PAAC showed their best AUC using RF models with a window size of 33. Details of the performance are shown in Table 1. Importantly, the models were only tested after the models were chosen. In other words, the test results did not play any role in the model selection.

**Table 1:** The performance of tenfold cross validation and independent testing for our proposed hybrid model in mammalian proteins. Since the hybrid model with RF models did not perform well, the final hybrid model did not incorporate them.

Feature	Dimension	Window size	Model	ACC	SEN	SPE	MCC	AUC
One hot encoding	30 × 21	31	CNN	0.713	0.712	0.714	0.233	0.784
AAINDEX	32 × 46	33	CNN	0.730	0.544	0.743	0.147	0.741
PSSM	33 × 20	33	CNN	0.707	0.707	0.707	0.216	0.775
AAC	21	23	RF	0.620	0.598	0.623	0.143	0.654
PAAC	34	17	RF	0.624	0.612	0.628	0.210	0.671
**Tenfold cross validation** Ensemble with NN (without RF models)				0.764	0.653	0.661	0.174	0.742
**Independent testing** Ensemble with NN (without RF models)				0.866	0.910	0.864	0.480	0.943

*PSSM* position specific scoring matrix, *AAC* amino acid composition, *PAAC* pseudo-amino acid composition, *CNN* convolutional neural network, *RF* random forest, *NN* neural network, *ACC* accuracy, *SEN* sensitivity, *SPE* specificity, *MCC* Matthews correlation coefficient, *AUC* area under the receiver operating characteristic curve.

The performance of our proposed hybrid model in mammalian proteins is shown in Table 1. After determining the best window size of each feature and the aggregation methods, we used a tenfold cross-validation method to compare the performance after the addition of more features. AAINDEX, PSSM, and One hot encoding were found to result in the best AUC. The performance of our proposed hybrid model in plant proteins is shown in Table 2. The ensemble model resulted in a great improvement.

**Table 2:** The performance of tenfold cross validation and independent testing for our proposed hybrid model in plant proteins.

Feature	Dimension	Window size	Model	ACC	SEN	SPE	MCC	AUC
One hot encoding	32 × 21	33	CNN	0.598	0.572	0.600	0.095	0.635
AAINDEX	26 × 30	27	CNN	0.637	0.577	0.642	0.121	0.673
PSSM	31 × 20	31	CNN	0.614	0.571	0.617	0.103	0.647
AAC	20	39	RF	0.654	0.632	0.656	0.161	0.720
PAAC	34	39	RF	0.661	0.633	0.663	0.166	0.718
**Tenfold cross validation** ensemble with SVM				0.660	0.653	0.661	0.174	0.742
**Independent testing** ensemble with SVM				0.691	0.682	0.692	0.195	0.772

*PSSM* position specific scoring matrix, *AAC* amino acid composition, *PAAC* pseudo-amino acid composition, *CNN* convolutional neural network, *RF* random forest, *NN* neural network, *ACC* accuracy, *SEN* sensitivity, *SPE* specificity, *MCC* Matthews correlation coefficient, *AUC* area under the receiver operating characteristic curve.

### Comparison with other existing malonylation site prediction tools in terms of predictive performance

**Figure 6:**
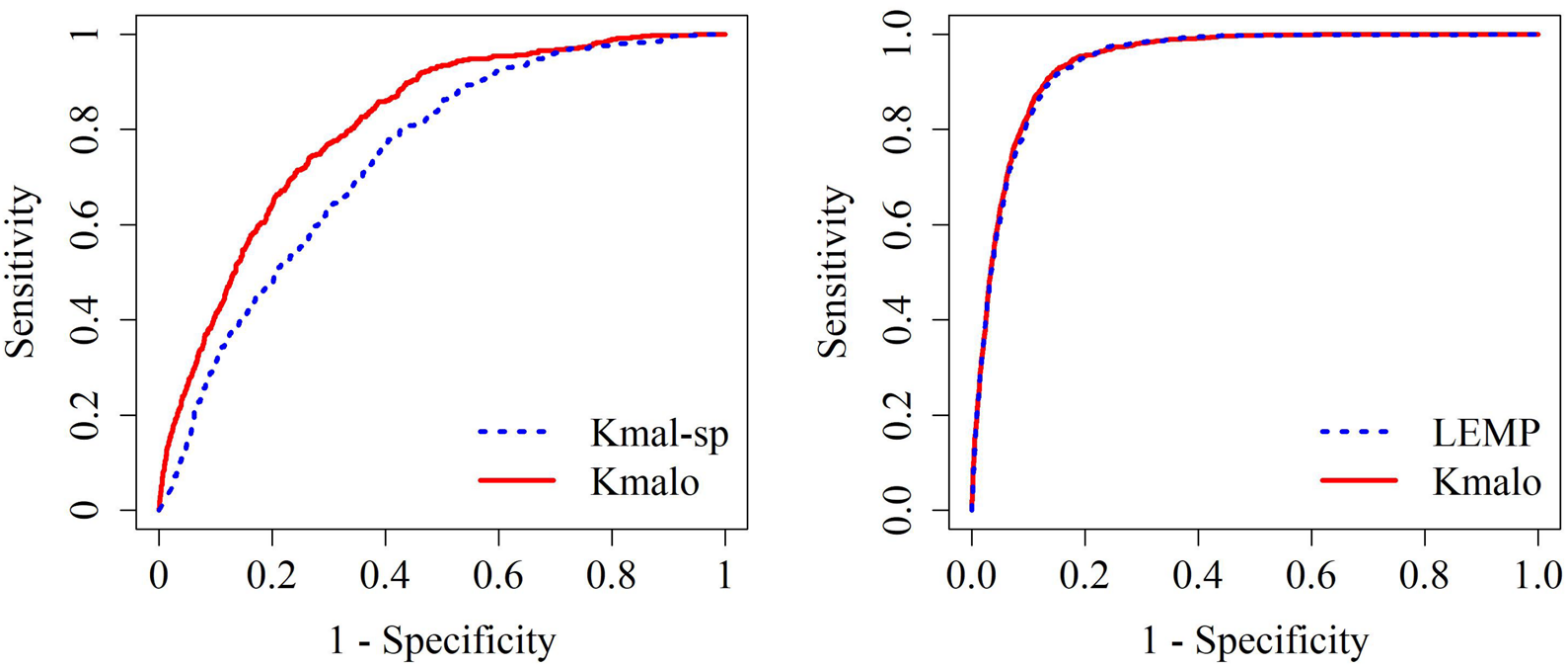
ROC curves on the independent testing for comparing with Kmal-sp (left) and LEMP (right).

We compared our model with other proposed computational prediction models. Table 3 and Fig. 6 demonstrates the comparative results. It should be noted that the peptides were removed from the independent dataset if the peptides were in the training dataset of the proposed computational prediction models. In total, 19,212 (683 positive sites, 18,529 negative sites) and 20,312 (1,251 positive sites, 19,061 negative sites) peptides were identified for the Kmal-sp^16^ *H. sapiens* model and LEMP^22^, respectively. These results indicate that the model proposed here was comparable to existing other tools.

**Table 3:** The comparisons of our model with Kmal-sp amd LEMP for predicting malonylation sites in mammalian proteins, respectively.

Tool	TP	FN	ACC	SEN	SPE	Time consuming
	FP	TN				
Kmal-sp	270	190	0.597	0.587	0.598	Around 2 days
	4,138	6,151				
Kmalo (proposed)	303	157	0.674	0.659	0.675	Within minutes
	3,349	6,940				
LEMP	1,130	121	0.862	0.903	0.860	Within minutes
	2,672	16,389				
Kmalo	1,138	113	0.866	0.910	0.864	Within minutes
	2,600	16,461				

*TP* true positive, *FP* false positive, *FN* false negative, *TN* true negative, *ACC* accuracy, *SEN* sensitivity, *SPE* specificity.

### Web interface of Kmalo

A web-based tool, Kmalo, has been developed to perform the prediction of protein malonylation sites. Users can predict malonylation sites in either mammalian or plant proteins. After submitting a protein in FASTA format via uploading a file or pasting the sequences into the tool, users will receive a job ID with which they can retrieve their results once the prediction process is finished. The results can be conveniently copied, printed directly, or downloaded in several formats, including CSV, XMSL, PDF. Snapshots of the tool’s website are shown in Supplementary Fig. S3.

## Discussions and conclusion

An increasing amount of studies are currently working towards improving our understanding of the mechanism of protein lysine malonylation. The role of this post-translational modification in a wide range of cell functions has drawn the attention of many research groups. In certain biological processes, malonylation is associated with the development of disease. However, laboratory experiments for the validation of malonylation sites are often time-consuming and expensive. In this study, we proposed a machine learning-based methodology for the prediction of malonylation sites, in hopes of reducing the economic and temporal efforts required by traditional methods. To obtain potential hidden information in the sequences, we applied a deep learning-based methodology to help extract interactive information from the evolutionary information, physicochemical properties, or simply the protein fragments, which were encoded by PSSM, AAindex, and one hot vector. Then, the two commonly used features for the identification of PTM sites, AAC, and PAAC were combined with the extracted information to obtain the final features.

Some previous studies have suggested building a malonylation sites prediction model for mammals using mouse and human proteins. Taherzadeh et al*.* proposed three machine learning-based models for predicting malonylation sites in humans, mice, and bacteria separately^15^. They used mouse proteins to test the model trained using human proteins, and used human proteins to test the model trained using mouse proteins. Both resulted in comparatively similar performances. This suggested that malonylation in mouse and human proteins have similar physicochemical mechanisms, which is consistent with the feature analyses shown in Fig. 2, Fig. 3 and Supplementary Fig. S1. Therefore, we constructed a predictor for the identification of malonylation sites using both mouse and human proteins in a single model.

Recent studies have revealed that malonylation sites impact the functional processes not only in mouse and human cells, but also in plant cells^10,11^. In fact, this study is the first to build a computational method for the identification of malonylation sites in plant proteins. We found that a complicated deep learning-based approach could lead to an overfitting problem. This could be due to the limited amount of data. As such, we modified our framework to make it more suitable for training only a small amount of data. After a series of experiments, we discovered that assembling the prediction results from the models trained by different features resulted in a robust model. Compared to the majority votes strategy, when combined with another SVM model, the former ensemble method resulted in a better performance.

In this study, we constructed hybrid models combining CNN and machine learning algorithms, including RF and SVM, in order to predict malonylation sites in mammals and plants, respectively. The competitive performance compared to the existed tools showed that the proposed hybrid scheme indeed generated useful information from raw protein sequence data. Therefore, the framework we proposed is expected to inspire others to develop novel computational methods on the related issues.

## Materials and methods

We created a useful identification system for the identification of malonylation sites, represented by a flow chart of steps, as shown in Supplementary Fig. S4,. Detailed explanations for each process described in the chart are provided in the following sections.

### Dataset preparation

In this study, we collected the mammalian proteins from protein lysine modifications database (PLMD)^32^ and LSTM-based ensemble malonylation predictor (LEMP)^22^. PLMD is an online database consisting of integrated protein lysine modifications, which includes 5,013 and 4,390 validated malonylation sites from 1841 *H. sapiens* and 1,466 M*. musculus* proteins, respectively. LEMP is a newer web tool and so far contains 5,288 malonylation sites and 88,636 non-malonylation sites for the prediction of malonylation sites in mammalian proteins. On the other hand, Liu et al.^10^ derived plant proteins from 342 malonylation sites in 233 T*. aestivum* proteins.

In order to prevent potential bias, a cluster database at a high identity with tolerance (CD-HIT)^33^ was used to reduce the redundancy at the cutoff threshold of 40% sequence identity. Then, the proteins were segmented into a fixed-length peptide fragment with a lysine (K) located at the center, denoted as:

$$ R_{ - n} R_{ - (n - 1)} \ldots R_{ - 2} R_{ - 1} KR_{1} R_{2} \ldots R_{n - 1} R_{n} , $$

where *R* is any of the 20 amino acids, and n represents the distance between the residue and the central *K*. The number of residues upstream and downstream of the center remained equal. In other words, the window size of each peptide was 2*n* + 1. If the length of the upstream peptide or downstream peptide was less than n, the dummy residue “*X*” would be used to fill the lacking residues. In this study, we considered window sizes from 15 to 35 (*n* = 7, 8, …, 16, 17). Then, we used CD-HIT-2D on positive and negative data to eliminate redundancy. Since the mammalian peptides collected from LEMP were processed with a window size 31, UniProt^34^ was employed to map the peptides to determine their residues for window sizes 33 and 35. Therefore, the mammalian proteins were composed of LEMP and the processed PLMD data; the redundant peptides were removed. After processing, 80% of the data were randomly selected to form the training dataset for the construction of the prediction model of the malonylation sites in mammalian peptides, and the remaining 20% of the data were selected for an independent testing dataset. Similarly, the same steps were used to process the wheat peptides in PLMD data. We randomly separated the wheat data with a ratio of 7:3 for training and test data for the prediction of malonylation sites in plants. Related works suggest a window size around 25 is suitable for predicting malonylation sites in mammalian proteins. However, little related works have suggested that a varying window sizes for the development of a malonylation site prediction model in plants. Consequently, we considered window sizes ranging from 11 to 39, a wider range than that was considered for mammalian peptides. The experimental datasets are summarized in Table 4 and Supplementary Fig. S5,.

**Table 4:** The number of malonylation and non-malonylation sites used in this study.

Species	Training set		Testing set		Independent testing set	
	Positive	Negative	Positive	Negative	Positive	Negative
*H. sapiens or* *M. musculus*	5,006	76,264	1,252	19,066	460^+^	10,289^+^
					1,251^#^	19,061^#^
*T. aestivum*	196	2,394	82	1,195	82	1,195

“ + ” means the testing set for Kmal-sp tool; “#” means the testing for LEMP tool.

### Features extraction

Amino acid composition (AAC) encoding calculates the frequency of the residues (20 standard amino acids and one dummy amino acid “*X*”) surrounding the modification sites and has been widely used in various prediction works^14^. Here, the site itself is not counted. As a result, each segment will be encoded as a 21-dimention vector.

Additionally, one hot encoding was used to express the sequence features. Specifically, the peptide was encoded as a two-dimensional matrix. The rows of the matrix were the amino acids in the peptide, and the columns were the 21 amino acids (with 1 dummy amino acid “*X*”). Every position in each row was filled with “0”, except the one that corresponds to the amino acid in the column, which was filled with “1”^35^ . This encoding method has been applied in many kinds of PTM site prediction^19,20^.

Pseudo-amino acid composition (PAAC) encoding was considered in this study. This group of descriptors was first proposed by Shen and Chou (2008)^36^. iFeature^37^ in Python was used to generate one of the encoding methods. The descriptor was used to integrate the information of the original hydrophobicity values, the original hydrophilicity values, and the original side chain masses of the 20 natural amino acids with the sequence-order information. As a result, PAAC not only considered the most adjacent residues, but also the adjacent plus λ residues. In this study, λ = 13.

The AAindex database^38^ collects indices of the representing physicochemical properties of amino acids. In this study, we used iFeature^37^ to encode each sample peptide. For each sample, the amino acid at each position was represented by 531 physicochemical properties, such that the vector of each encoding peptide was 531 × (*L*–1) dimensional (where *L* is the window size of the peptide; only the upstream and downstream were employed). Then, a feature-selection method was applied to remove the redundant properties and obtain the optimal physicochemical property sets. iFeature was used to calculate the information gain of our AAindex-based feature, which was then added to the value of information gain for each physicochemical property at each position. With the highest summed-up value of information gain, 46 physicochemical properties were selected for mammalian proteins and 30 physicochemical properties for plant proteins. After reshaping the features, a two-dimensional matrix with 46 (or 30) columns and (*L*–1) rows for each sample fragment was obtained.

Position-specific scoring matrix (PSSM) profiles have been used in several related works^14–16^. In this study, PSI-BLAST was performed with a default E-value cutoff in three iterations. The size of the profile was *L* × 20, where *L* represents the window size and 20 denotes the number of the standard amino acids. It should be noted that the PSSM profile of the fragment was directly used as the input of the deep learning model, without reshaping.

### Hybrid model construction

The proposed hybrid models are composed of two parts. In the first part, a series of experiments were performed to develop the best prediction model for each single-feature category. Specifically, RF, SVM, and CNN were implemented for the one hot encoding features, including the AAC, PAAC, AAindex, and PSSM profiles, respectively. It should be noted that each model was trained with peptides ranging from window size 15 to 35 in order to determine the proper length. RF models were constructed using the Python package “Scikit-learn”^39^ , where the number of decision trees was 500. Radial basis function kernel was chosen as the default kernel function for our SVM models. The window size of the optimal model with the highest area under the receiver operating characteristic curve (AUC) score was selected as the most suitable window size. A CNN model including two rounds of the convolutional layers and max pooling layers followed by three fully connected layers was designed. At this stage, 5 single-feature models for mammalian proteins and 5 single-feature models for plant proteins were obtained. Herein, the ‘Tensorflow’ package in Python was used.

The second step involved the aggregation of the separated models trained with different features in order to make a final prediction. Three methodologies for aggregation were used: another neural network, SVM, and majority votes. For the mammalian proteins, considering the CNN model as a feature extractor and combined with the raw AAC, the feeding of the PAAC feature into another neural network obtained the best AUC. On the other hand, for the plant proteins, feeding the positive probability of each feature into a SVM model resulted in the best performance. The schemes of proposed model for mammalian and plant proteins are shown in Supplementary Fig. S6, and Supplementary Fig. S7,, respectively. The cutoff values are 0.046 and 0.195 for mammalian and plant models, respectively.

### Metrics of model evaluation

The following metrics were used to evaluate the performance of our models: sensitivity (SEN), specificity (SPE), accuracy (ACC), and Matthews correlation coefficient (MCC). The detailed definitions of these metrics are given below.

$$ SEN = \frac{TP}{{TP + FN}}, $$

$$ SPE = \frac{TN}{{FP + TN}}, $$

$$ ACC = \frac{TP + TN}{{TP + TN{ + }FP + FN}}, $$

$$ MCC = \frac{(TP \times TN) - (FP \times FN)}{{\sqrt {(TP{ + }FP) \times (TP{ + }FN) \times (FP{ + }TN) \times (TN{ + }FN)} }}, $$

where TP denotes the true positives and refers to the number of positive labels that were correctly predicted by the classifier, TN denotes the true negatives and refers to the number of negative labels that were correctly predicted by the classifier, FP denotes the false positives and refers to the number of positive labels that were incorrectly predicted by the classifier, and FN denotes the false negatives and refers to the number of negative labels that were incorrectly predicted by the classifier. Additionally, the area under the receiver operating characteristic curve (AUC) was also considered in this study. It should be noted that a receiver operating characteristic curve (ROC) is a widely used visual tool for comparing predicted performances. It is able to demonstrate the trade-off between the true positive rate (TPR) and the false positive rate (FPR) at various threshold settings. Therefore, the AUC is frequently used when evaluating the overall predictive performance of a wide range of biological systems.

### Development of the web-based prediction tool

The web-based prediction tool was mainly written in HyperText Markup Language (HTML), Cascading Style Sheets (CSS), JavaScript (JS), and HyperText Preprocessor (PHP). When a user submits data, this data will be saved as a text file named with a combination of the date, time, and five random characters or numbers in an automatically created folder in the server computer. A series of feature extractions will be performed and every feature will be saved as a text file in the same folder for further use. For the same reason, the prediction result will also be saved as a text file. If the user waits on the same page after having submitted their data, they will be automatically redirected to the results page. Otherwise, the user can use the job ID, with the same name as the automatically-saved input file, to retrieve his or her results.

## Supplementary information

Supplementary file1 (DOCX 2063 kb)

## Data Availability

The datasets used and analyzed during the current study are available from the corresponding authors on reasonable request.
