# Supplementary material for: Incorporating hybrid models into lysine malonylation sites prediction on mammalian and plant proteins
Source: Sci Rep. 2020 Jun 29;10:10541. doi: 10.1038/s41598-020-67384-w (PMC7324624; doi:10.1038/s41598-020-67384-w)
Supplement: Supplementary file 1 — Supplementary file1 (DOCX 2063 kb) [file 41598_2020_67384_MOESM1_ESM.docx]

Incorporating Hybrid Models into Lysine Malonylation Sites Prediction on Mammalian and Plant Proteins

Chia-Ru Chung^1^, Ya-Ping Chang^1^, Yu-Lin Hsu^1^, Siyu Chen^2^, Li-Ching Wu^3^, Jorng-Tzong Horng^1,4,*^ and Tzong-Yi Lee^2,5,*^

*^1^Department of Computer Science and Information Engineering, National Central University, Taoyuan 32001, Taiwan*

*^2^School of Life and Health Sciences, The Chinese University of Hong Kong, Shenzhen 518172, China*

*^3^Department of Biomedical Sciences and Engineering, National Central University, Taoyuan 32001, Taiwan*

*^4^Department of Bioinformatics and Medical Engineering, Asia University, Taichung 41359, Taiwan*

*^5^Warshel Institute for Computational Biology, The Chinese University of Hong Kong, Shenzhen 518172, China*

*To whom correspondence should be addressed: TY Lee: [leetzongyi@cuhk.edu.cn](mailto:leetzongyi@cuhk.edu.cn) and JT Horng: [horng@db.csie.ncu.edu.tw](mailto:horng@db.csie.ncu.edu.tw)

Email addresses:

CR Chung: [jjrchris@g.ncu.edu.tw](mailto:jjrchris@g.ncu.edu.tw)

YP Chang: yp.c9797@gmail.com

SY Chen: [117010024@link.cuhk.edu.cn](mailto:117010024@link.cuhk.edu.cn)

YL Hsu: 103201525hsu@gmail.com

LC Wu: [nculcwu@gmail.com](mailto:nculcwu@gmail.com)

**Supplementary Materials**


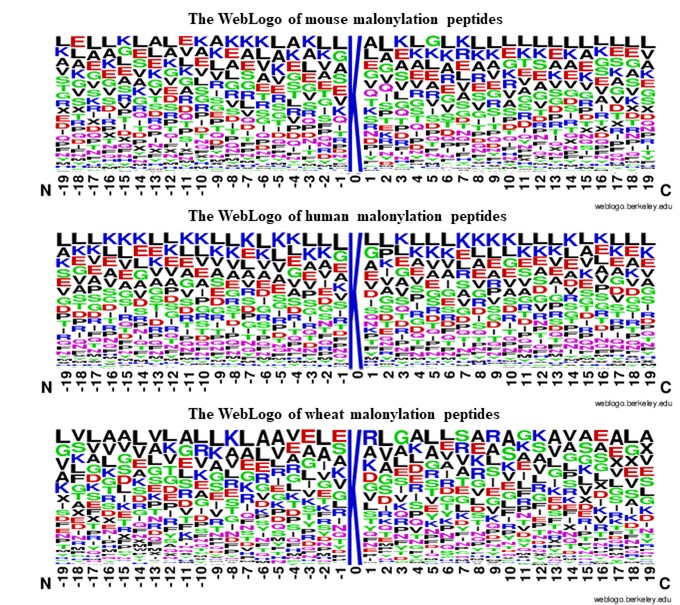


**Figure S1.** The WebLogo of the malonylation peptides for three organisms.


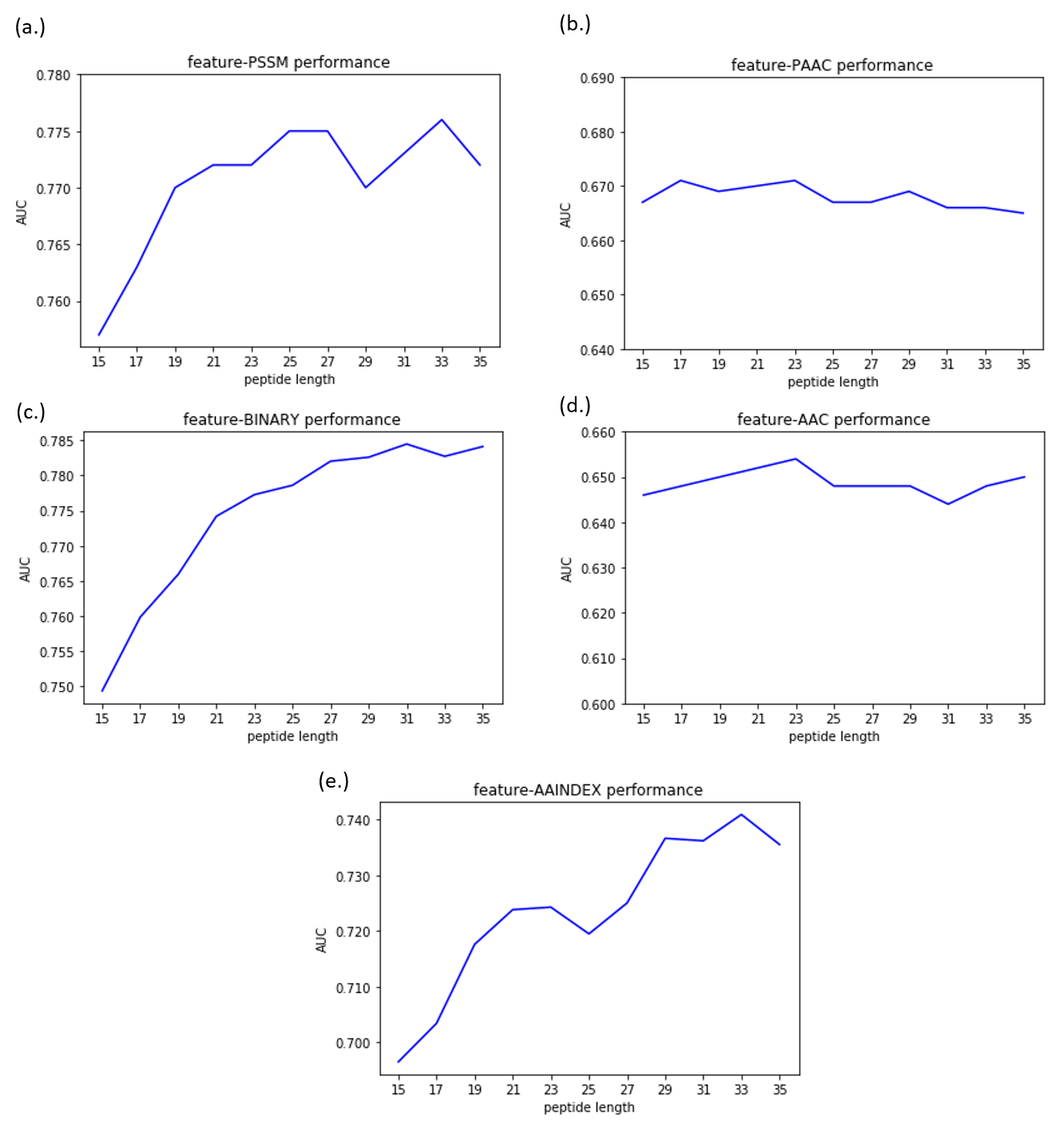


**Figure S2.** The AUCs for different window sizes in each feature. (a.) PSSM trained by CNN, (b.) PAAC trained by RF, (c.) One hot vector trained by CNN, (d.) AAC trained by RF, and (e.) AAINDEX trained by CNN.


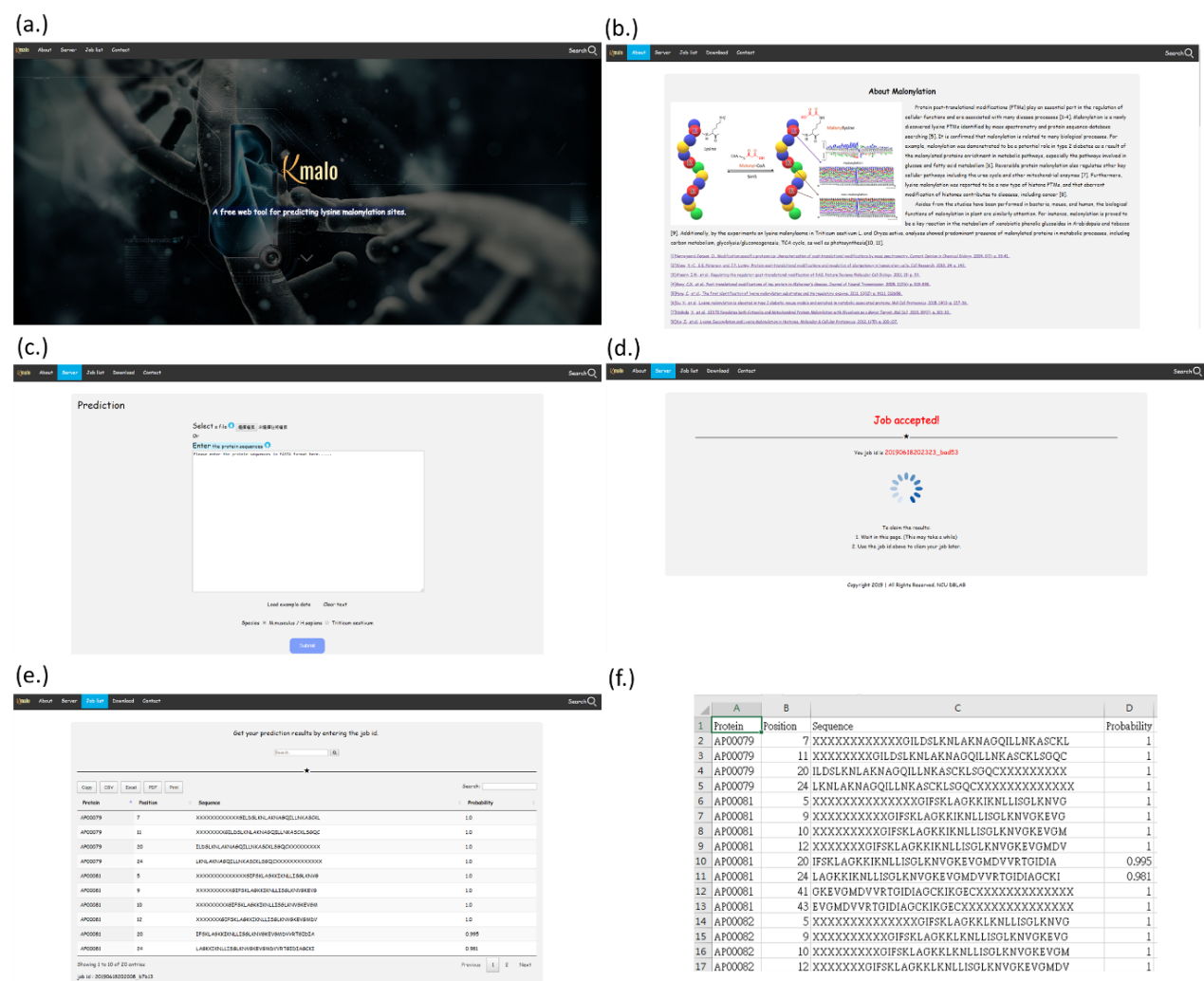


**Figure S3.** Snapshots of Kmalo. (a.) the welcome page, (b.) information page of malonylation, (c.) web tool page, (d.) the page after the user submit the sequences, (e.) the page showing the prediction results, and (f.) the downloaded csv file of the results.


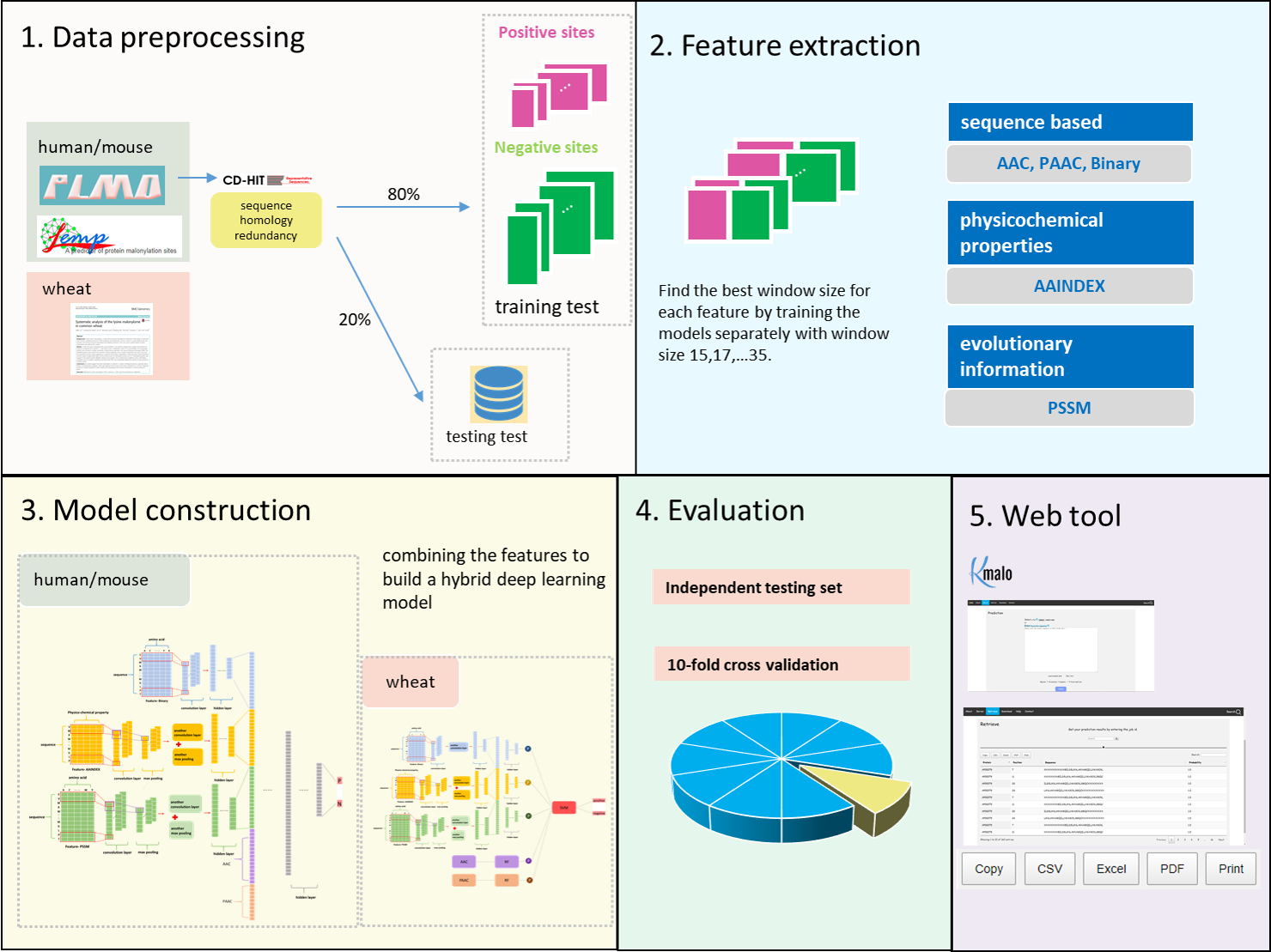


**Figure S4.** Flow chart of this study.


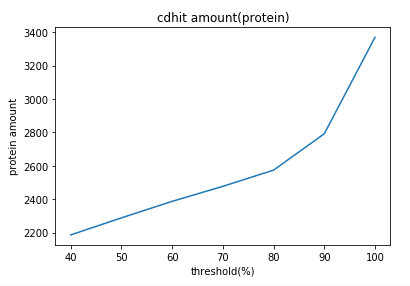


**Figure S5.** The number of the protein after performing CD-HIT with different thresholds in mammalian proteins which obtained from PLMD [23].


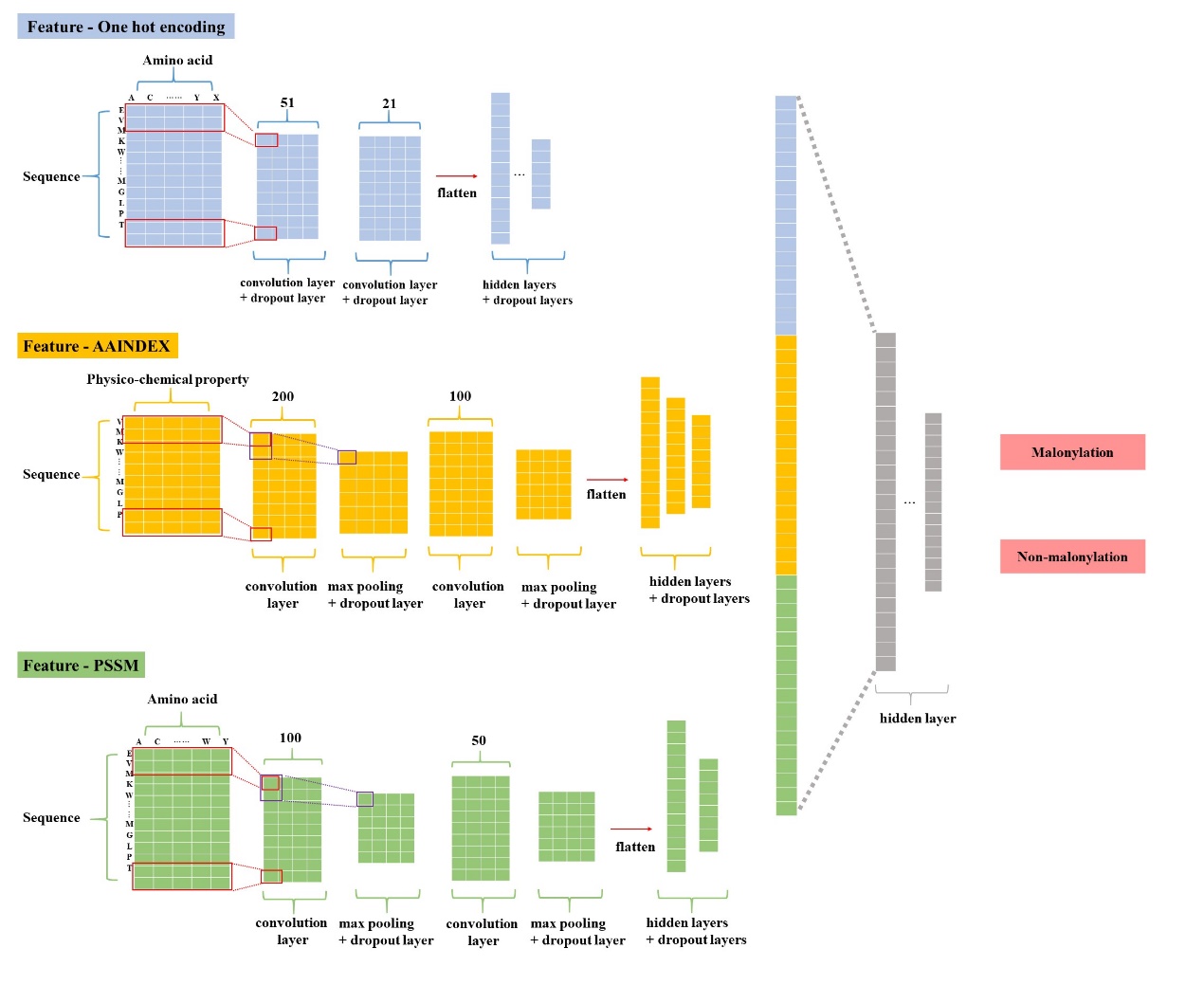


**Figure S6.** The deep learning architecture for identifying malonylation sites on mammalian proteins. Three CNNs trained from sequence, AAindex, and PSSM individually are integrated into secondary neural network for classifying between malonylated and non-malonylated sequences.


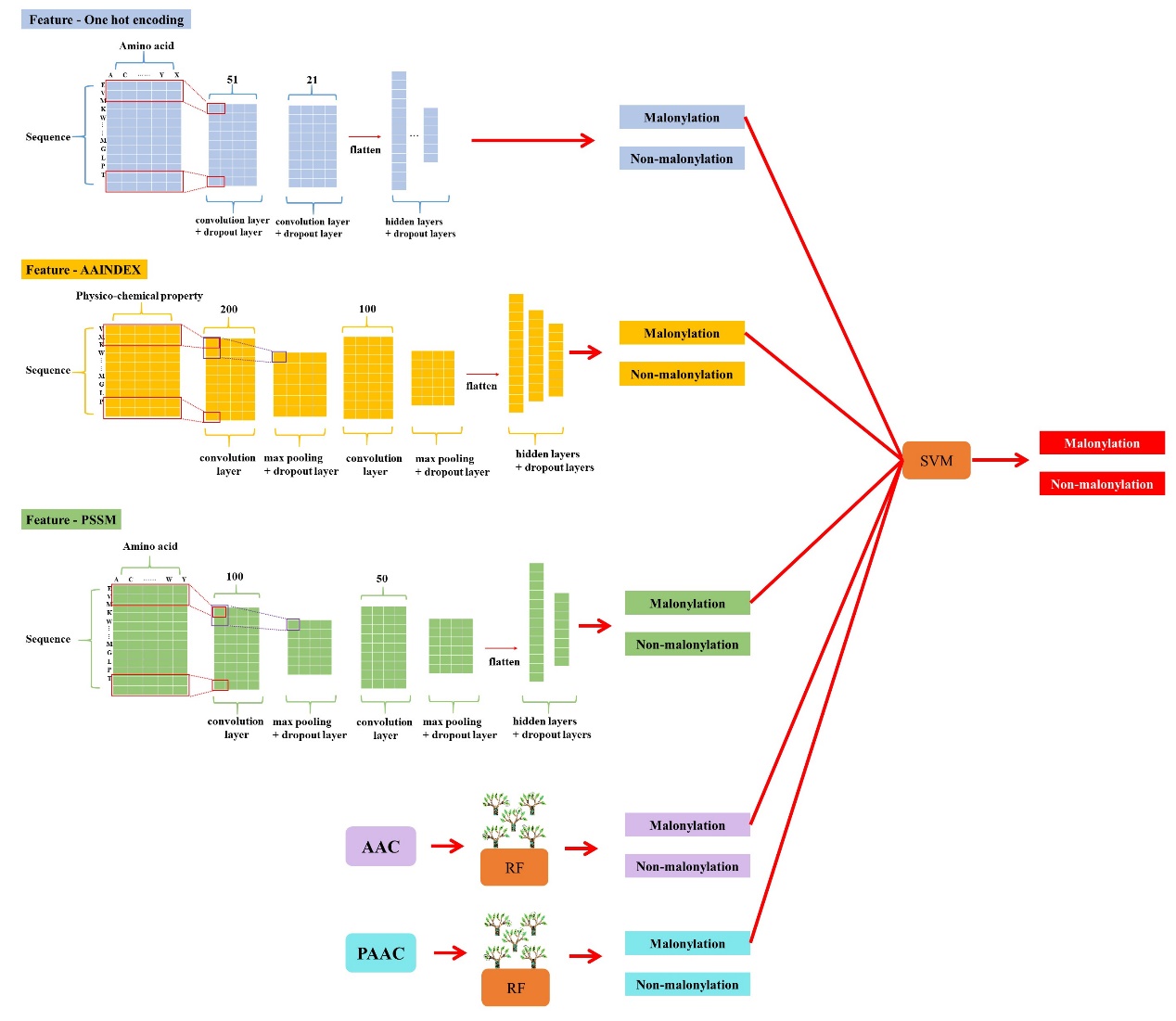


**Figure S7.** The deep learning architecture for identifying malonylation sites on plant proteins. In the first step, sequence feature, AAindex, and PSSM were individually trained by CNN. AAC and PAAC were trained by RF. In the second step, the predicted malonylation probabilities were integrated into SVM for classifying between malonylated and non-malonylated sequences.

**Table S1**. Top ten important feature list for each feature category in mammalian proteins.

| Category | Feature name | Pearson’s correlation coefficients |
| --- | --- | --- |
| AAC | E | -0.052 |
|  | G | 0.047 |
|  | V | 0.031 |
|  | S | -0.030 |
|  | A | 0.025 |
|  | W | -0.020 |
|  | K | 0.014 |
|  | I | 0.013 |
|  | R | 0.010 |
|  | Q | -0.010 |
| PAAC | PAAC.λ1 | 0.065 |
|  | PAAC.E | -0.056 |
|  | PAAC.G | 0.041 |
|  | PAAC.S | -0.031 |
|  | PAAC.λ2 | 0.027 |
|  | PAAC.λ6 | 0.026 |
|  | PAAC.λ3 | 0.025 |
|  | PAAC.λ13 | 0.023 |
|  | PAAC.W | -0.023 |
|  | PAAC.V | 0.021 |
| PSSM | Pos.13.G | -0.045 |
|  | Pos.13.K | 0.043 |
|  | Pos.13.W | -0.043 |
|  | Pos.13.I | -0.042 |
|  | Pos.14.R | -0.040 |
|  | Pos.13.Y | -0.040 |
|  | Pos.14.K | -0.039 |
|  | Pos.13.F | -0.039 |
|  | Pos.13.R | 0.038 |
|  | Pos.13.Q | 0.037 |
| One hot encoding | One hot encoding.F344 | 0.036 |
|  | One hot encoding.F348 | -0.028 |
|  | One hot encoding.F323 | 0.025 |
|  | One hot encoding.F280 | -0.025 |
|  | One hot encoding.F338 | -0.023 |
|  | One hot encoding.F474 | 0.022 |
|  | One hot encoding.F260 | 0.022 |
|  | One hot encoding.F386 | 0.021 |
|  | One hot encoding.F364 | -0.021 |
|  | One hot encoding.F406 | -0.021 |
| AAindex | SeqPos.18.JANJ780101 | -0.041 |
|  | SeqPos.18.FAUJ880104 | -0.041 |
|  | SeqPos.18.JANJ780103 | -0.040 |
|  | SeqPos.18.RICJ880113 | -0.040 |
|  | SeqPos.18.LEVM760105 | -0.039 |
|  | SeqPos.18.RADA880107 | 0.037 |
|  | SeqPos.18.CHOC760102 | -0.037 |
|  | SeqPos.18.NAKH900110 | 0.036 |
|  | SeqPos.18.RADA880106 | -0.035 |
|  | SeqPos.18.GUYH850105 | -0.035 |

**Table S2**. Top ten important feature list for each feature category in plant proteins.

| Category | Feature name | Pearson’s correlation coefficients |
| --- | --- | --- |
| AAC | X | 0.167 |
|  | R | 0.112 |
|  | D | -0.095 |
|  | W | -0.066 |
|  | E | -0.053 |
|  | L | -0.038 |
|  | M | -0.032 |
|  | P | -0.032 |
|  | A | 0.029 |
|  | C | 0.026 |
| PAAC | PAAC.X | 0.167 |
|  | PAAC.R | 0.112 |
|  | PAAC.D | -0.103 |
|  | PAAC.W | -0.068 |
|  | PAAC.E | -0.060 |
|  | PAAC.λ6 | 0.059 |
|  | PAAC.λ2 | 0.050 |
|  | PAAC.λ5 | 0.049 |
|  | PAAC.λ3 | 0.049 |
|  | PAAC.L | -0.049 |
| PSSM | Pos.18.R | 0.067 |
|  | Pos.25.R | 0.062 |
|  | Pos.8.K | 0.061 |
|  | Pos.26.R | 0.058 |
|  | Pos.18.K | 0.054 |
|  | Pos.8.R | 0.054 |
|  | Pos.15.S | 0.050 |
|  | Pos.25.K | 0.049 |
|  | Pos.25.Q | 0.048 |
|  | Pos.2.W | 0.047 |
| One hot encoding | One hot encoding.F147 | 0.129 |
|  | One hot encoding.F168 | 0.129 |
|  | One hot encoding.F546 | 0.120 |
|  | One hot encoding.F126 | 0.117 |
|  | One hot encoding.F105 | 0.117 |
|  | One hot encoding.F525 | 0.116 |
|  | One hot encoding.F189 | 0.116 |
|  | One hot encoding.F504 | 0.116 |
|  | One hot encoding.F567 | 0.105 |
|  | One hot encoding.F42 | 0.096 |
| AAindex | SeqPos.14.ROSM880102 | 0.065 |
|  | SeqPos.21.ROSM880102 | 0.059 |
|  | SeqPos.14.RADA880102 | -0.056 |
|  | SeqPos.21.RADA880105 | -0.055 |
|  | SeqPos.22.RADA880105 | -0.052 |
|  | SeqPos.2.QIAN880102 | -0.052 |
|  | SeqPos.14.NADH010102 | -0.049 |
|  | SeqPos.25.SNEP660103 | 0.048 |
|  | SeqPos.13.ROSM880102 | 0.048 |
|  | SeqPos.24.MCMT640101 | 0.046 |
